# Supplementary figures and images for: Defense-Related Enzyme Activities and Metabolomic Analysis Reveal Differentially Accumulated Metabolites and Response Pathways for Sheath Blight Resistance in Rice
Source: Plants (Basel). 2024 Dec 19;13(24):3554. doi: 10.3390/plants13243554 (PMC11677778; doi:10.3390/plants13243554)

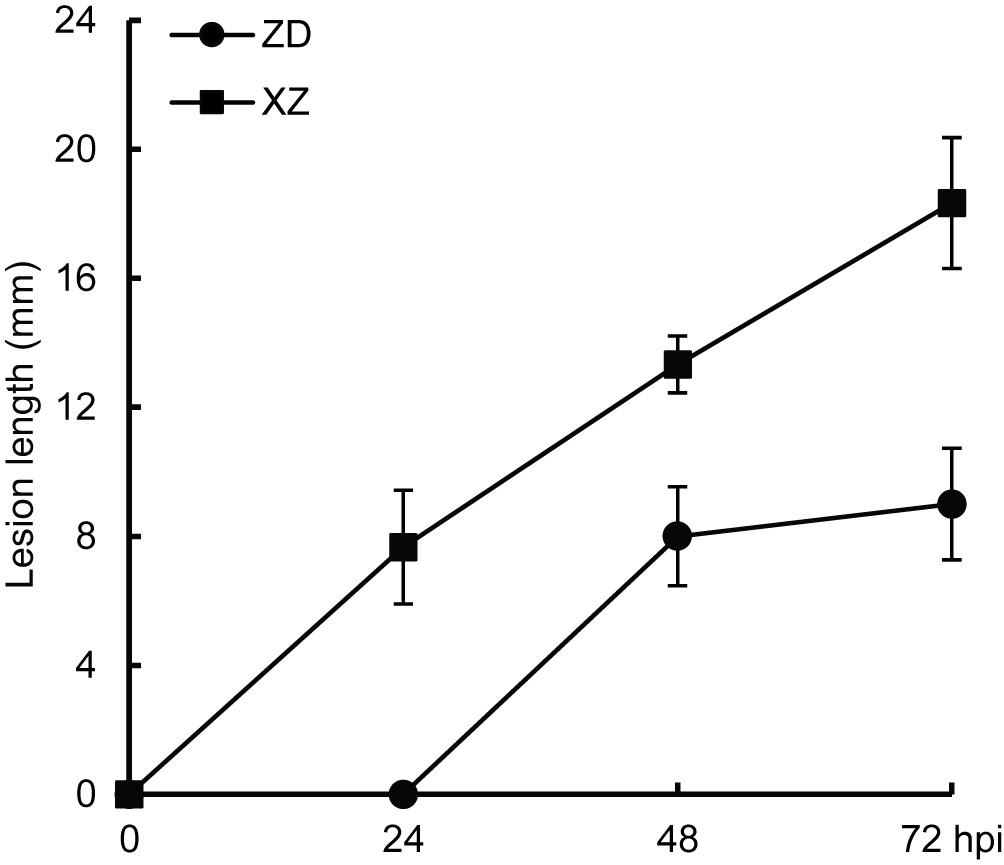

Supplement: Supplementary file 1 [file plants-13-03554-s001.zip › Figure S1.tif]
